# Publisher Correction: Structure and activation of the RING E3 ubiquitin ligase TRIM72 on the membrane

**DOI:** 10.1038/s41594-024-01429-w

**Published:** 2024-10-28

**Authors:** Si Hoon Park, Juhyun Han, Byung-Cheon Jeong, Ju Han Song, Se Hwan Jang, Hyeongseop Jeong, Bong Heon Kim, Young-Gyu Ko, Zee-Yong Park, Kyung Eun Lee, Jaekyung Hyun, Hyun Kyu Song

**Affiliations:** 1 Department of Life Sciences, Korea University, Seoul, South Korea; 2 School of Life Sciences, Gwangju Institute of Science and Technology, Gwangju, South Korea; 3 Center for Electron Microscopy Research, Korea Basic Science Institute, Cheongju-si, South Korea; 4 Advanced Analysis Center, Korea Institute of Science and Technology, Seoul, South Korea; 5 School of Pharmacy, Sungkyunkwan University, Suwon, South Korea; 6 Present Address: Friedrich Miescher Institute for Biomedical Research, Basel, Switzerland; 7 Present Address: CSL Seqirus, Waltham, MA USA; 8 Present Address: Department of Pharmacology and Dental Therapeutics, School of Dentistry, Chonnam National University, Gwangju, South Korea

**Keywords:** Structural biology, Molecular biology

Correction to: *Nature Structural & Molecular Biology* 10.1038/s41594-023-01111-7, published online 28 September 2023.

In the version of the article initially published, in Fig. 3g, the label now reading “PS liposomes” originally read “PC liposomes” and has now been amended in the HTML and PDF versions of the article.

