# Supplementary material for: Structure and activation of the RING E3 ubiquitin ligase TRIM72 on the membrane
Source: Nat Struct Mol Biol. 2023 Sep 28;30(11):1695–706. doi: 10.1038/s41594-023-01111-7 (PMC10643145; doi:10.1038/s41594-023-01111-7)
Supplement: Supplementary file 1 — Supplementary Tables 1 and 2 and Figs. 1–9. [file 41594_2023_1111_MOESM1_ESM.pdf]

nature structural & molecular biology

Article https://doi.org/10.1038/s41594-023-01111-7

Structure and activation of the RING E3 ubiquitin ligase TRIM72 on the membrane

In the format provided by the authors and unedited

Supplementary Information

Structure and activation of the RING E3 ubiquitin ligase TRIM72 on the membrane

Si Hoon Park[1,a], Juhyun Han[1], Byung-Cheon Jeong[1,b], Ju Han Song[1,c], Se Hwan Jang[2], Hyeongseop Jeong[3], Bong Heon Kim[1], Young-Gyu Ko[1], Zee-Yong Park[2], Kyung Eun Lee[4], Jaekyung Hyun[5] and Hyun Kyu Song[1,*]

[1]*Department of Life Sciences, Korea University, 145 Anam-ro, Seongbuk-gu, Seoul 02841, South Korea*

[2]*School of Life Sciences, Gwangju Institute of Science and Technology, 123 Cheomdangwagi-ro, Buk-gu, Gwangju 61005, South Korea*

[3]*Center for Electron Microscopy Research, Korea Basic Science Institute, 161 Yeongudanji-ro, Ochang-eup, Cheongwon-gu, Cheongju-si, Chungcheongbuk-do 28119, South Korea*

[4]*Advanced Analysis Center, Korea Institute of Science and Technology (KIST), 5 Hwarang-ro 14-gil, Seongbuk-gu, Seoul 02792, South Korea*

[5]*School of Pharmacy, Sungkyunkwan University, 2066 Seobu-ro, Jangan-gu, Suwon 16419, South Korea*

*Correspondence: Hyun Kyu Song, Department of Life Sciences, Korea University, 145 Anam-ro, Seongbuk-gu, Seoul 02841, South Korea; Tel.: +82-2-3290-3457; Fax: +82-2-3290-3628; E-mail: hksong@korea.ac.kr

Supplementary Table 1. TRIM72 constructs used in this study.

Name	Construct	Description
***Human* TRIM72**		
WT	1-477	Selectively bound to microvesicles
Q57R	1-477, Q57R	Constitutively active mutation in ubiquitination
Q57R/L74R	1-477, Q57R/L74R	Mutation inactivating constitutive ubiquitination
ΔH_3	1-477, Deletion of 272-281	Defective mutation recognizing phosphatidylserine
K^2D	1-477, K460D/K462D	Defective mutation recognizing phosphatidylserine
R^3E	1-477, R368E/R369E/R371E	Defective mutation recognizing phosphatidylserine
GST-H_3-PRYSPRY	270-471	phosphatidylserine binding in a dimeric form
MBP-H_3-PRYSPRY	270-471	Defects in phosphatidylserine binding in a monomeric form
RBCC	7-268	Defective mutation recognizing phosphatidylserine
***Mouse* TRIM72**		
WT	2-477	Crystal structure (7.1 Å)
FL	7-470, C55S/C144S/K279H/K283H	Crystal structures (3.5 & 4.6 Å)
FL/C242S	7-470, C55S/C144S/C242S/K279H/K283H	Crystal structures (5.2 Å)
ΔRING	79-470, C55S/C144S/K279H/K283H	Crystal structures (2.75 & 3.28 Å)
RING	1-81	No effect on ubiquitination activity
2xRING	Linear fusion of 1-81 to 1-81	No effect on ubiquitination activity
RING+WT	Linear fusion of 1-81 to 1-477	No effect on ubiquitination activity
ΔH_3	2-477, Deletion of 272-281	Defective mutation recognizing phosphatidylserine
K^2D	2-477, K460D/K462D	Defective mutation recognizing phosphatidylserine

Supplementary Table 1. TRIM72 constructs used in this study (continued).

Name	Construct	Description
***Mouse* TRIM72**		
R^{3}E	2-477, R368E/R369E/R371E	Defective mutation recognizing phosphatidylserine
Q57R	2-477, Q57R	Constitutively active mutation
Q57R/E207R	2-477, Q57R/E207R	No effect on constitutive ubiquitination activity
Q57R/L74R	2-477, Q57R/L74R	Mutation inactivating constitutive ubiquitination
RING_Q57R	1-81, Q57R	Constitutively active mutation in ubiquitination
2×RING_Q57R	2xRING, Q57R	Constitutively active mutation in ubiquitination
G106R	2-477, G106R	Defective mutation in higher-order assemblies
S110R	2-477, S110R	Defective mutation in higher-order assemblies
M138A	2-477, M138A	Defective mutation in higher-order assemblies
M138R	2-477, M138R	Hyperactive mutation in higher-order assemblies
F274A/W277A	2-477, F274A/W277A	Defective mutation recognizing phosphatidylserine
***Rhesus* TRIM5α**		
RING$^{TRIM5\alpha}$	2-92	Refs. [1-4]
2×RING$^{TRIM5\alpha}$	2-82-GS-2-92	Refs. [2-4]

Supplementary Table 2. SAXS-derived data collection and structural parameters.

	Mouse TRIM72 WT	Mouse TRIM72 ΔRING
**Sample details**		
Organism	*Mus musculus*	*Mus musculus*
UniProt sequence ID (residues in construct)	Q1XH17 (2-477)	Q1XH17 (79-470$^{C144S/C242S/K279H/K283H}$)
Extinction coefficient [A_{280}, 0.1% (*w/v*)]	0.691	0.826
Mass from chemical composition (Da)	52,742	44,139
SEC-SAXS column	UPLC coupled with 5 x 150 mm Superdex™ 200 Increase 10/300 GL	
Loading concentration (mg mL^{-1})	8.2	13.0
Injection volume (µL)	240	240
Flow rate (mL min^{-1})	0.05	0.05
Solvent (solvent blanks taken from SEC flowthrough prior to elution)	25 mM Tris-HCl pH 8.0, 300 mM NaCl, 1 mM TCEP, and 1 mM DTT	
**SAXS data collection parameters**		
Instrument/data processing	Photon Factory BL-10C with PILATUS3 2M	
Wavelength (Å)	1.0	
Beam size (mm, FWHM, Vertical × Horizontal)	0.180 × 0.630	
Camera length (m)	3.012	
q measurement range (Å^{-1})	0.0050-0.2692	
Exposure time	Continuous 5-s data frame measurements with an interval period of 0.1 s	
Sample configuration	Path length of 1 mm and a pair of 20-µm quartz windows	
Column temperature (°C)	20	
Sample temperature (°C)	20	
**SAXS data reduction, analysis, and interpretation**		
Data reduction and solvent subtraction	*SAngler*, *CHROMIX* from *ATSAS* 3.0.1	
Extinction coefficient estimate	*ProtParam*	
Basic analyses: Guinier, *P(r)*, V_P	*PRIMUSqt* from *ATSAS* 3.0.1	
Shape/bead modeling	*DAMMIN via ATSAS* online (https://www.embl-hamburg.de/biosaxs/atsas-online/), *DAMSEL*, *DAMSUP*, *DAMAVER*, *SASRES*, *SUPCOMB* from ATSAS 3.0.1	
Atomic structure modeling	*SREFLEX via ATSAS* online, *CRYSOL via ATSAS* online	
3D graphic model representations	*PyMOL*	

Supplementary Table 2. SAXS-derived data collection and structural parameters (continued).

	Mouse TRIM72 WT	Mouse TRIM72 ΔRING
**Structural parameters**		
SASBDB ID	SASDK86	SASDK96
Guinier analysis		
$I(0)$ (cm^{-1})	0.0015 ± 0.000007	0.0021 ± 0.000005
R_g (Å)	68.56 ± 0.52	50.65 ± 0.20
q_{min} ($Å^{-1}$)	0.0079	0.0127
qR_g max (q_{min} = 0.0050 $Å^{-1}$)	1.21	1.23
Fidelity	0.57	0.64
P(r) analysis		
$I(0)$ (cm^{-1})	0.0015 ± 0.000005	0.0021 ± 0.000005
R_g (Å)	68.69 ± 0.33	52.36 ± 0.17
d_{max} (Å)	240	187
q range ($Å^{-1}$)	0.0079-0.1166	0.0126-0.1578
Total quality estimate (from *GNOM*)	0.48	0.53
Porod volume ($Å^{-3}$) (ratio Vp/calc. M, dimer)	234,209 (2.2)	142,315 (1.6)
Molecular mass analysis from (Da)		
Qp	173,145	101,738
MoW	144,715	102,921
Vc	121,306	92,290
Bayesian inference	146,800	97,500
**Atomic modeling**		
SREFLEX (with default parameters)		
Starting crystal structure	PDB ID: 7XYZ	
No. of rigid body domains	3	
χ^2 range	2.52-3.50	
No. of representative structures	25	

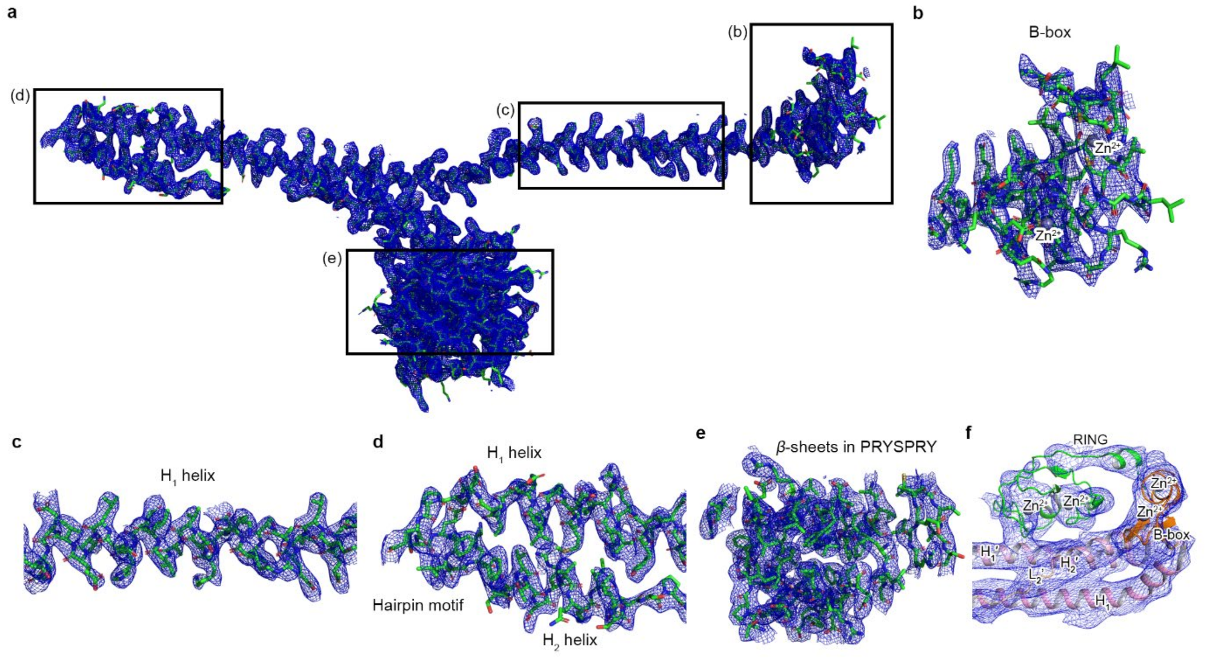

Supplementary Fig. 1| Electron density map of TRIM72 ΔRING.

a, Overall electron density map of the TRIM72 ΔRING monomer containing four representative boxed regions (b), (c), (d), and (e). **b-e**, Electron density maps of the B-box (**b**), H_1 helix (**c**), H_1 helix-hairpin motif-H_2 helix (**d**), and *β*-sheets in the PRYSPRY domain (**e**). The models and electron density maps were derived from the crystal structure of TRIM72 ΔRING (2.75 Å). **f**, Electron density map of the RING domain in the bent conformation of TRIM72 FL (4.6 Å). The 2Fo-Fc maps are contoured at 1.0 σ. The models are shown in stick representation with carbon (green), nitrogen (blue), oxygen (red), and sulfur (yellow) in Panels **a**-**e** and as a ribbon diagram in Panel **f**. Zinc atoms are represented as gray spheres.

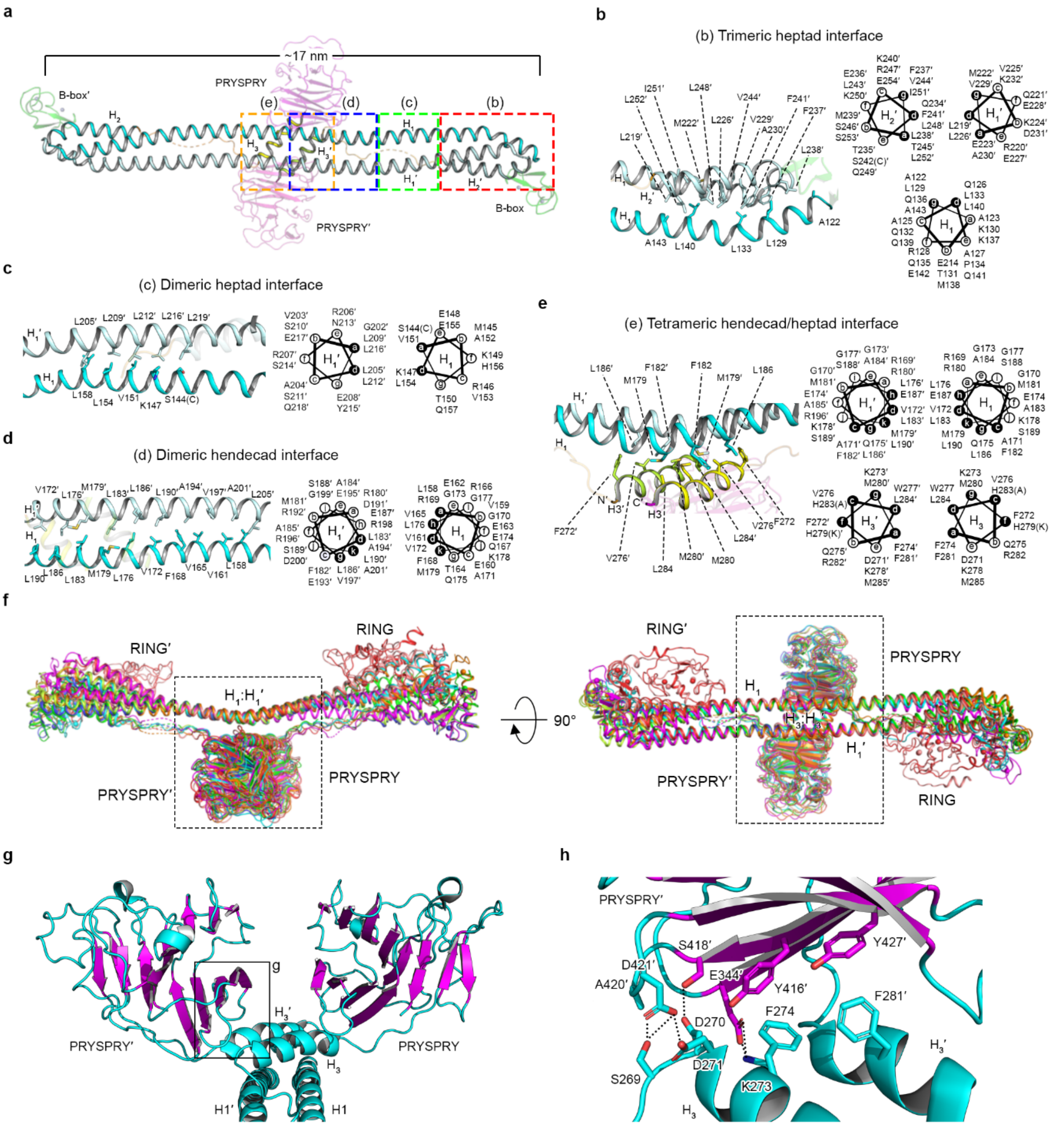

Supplementary Fig. 2| Structural analysis of the interface between CCDs.

a, Overall structure of the TRIM72 CCD shown as a ribbon diagram. CCD interactions are categorized as belonging to four regions: b (red), c (green), d (blue) and e (orange). The other protomer is labeled with prime (') symbols. **b-e,** CCD interfaces in the trimeric heptad interface (**b**), dimeric heptad interface (**b**), dimeric hendecad interface, (**d**) and four helical bundles of the heptad/hendecad interface (**e**). Each region is represented by ribbon (left) and helical wheel (right) diagrams (**b-e**). The black circled positions in the wheels are hydrophobic repeats buried

in the interface. The amino acid residues of mouse TRIM72 WT are labeled in both the ribbon and helical wheel diagrams. The mutated residues for crystallization are indicated in S144 (C), S242 (C), H279 (K) and H283 (A). The WT residues are described in parentheses. **f,** Superposition of eight determined crystal structures. The dotted line boxes indicate the structurally rigid part formed by the hendecad repeated region of H_1:H_1', the heptad repeated region of H_3:H_3', and the PRYSPRY pair. Note that the CCDs move in only one direction, while the peripheral CCD and B-box are very flexible. **g,** Orientation of two PRYSPRY domains maintained by interactions of the H_3 helices. **h,** Interface between the H_3 helices and PRYSPRY domain. Panel **h** shows a close-up view of the black box in **g**. All models were derived from the crystal structure of TRIM72 ΔRING (2.75 Å).

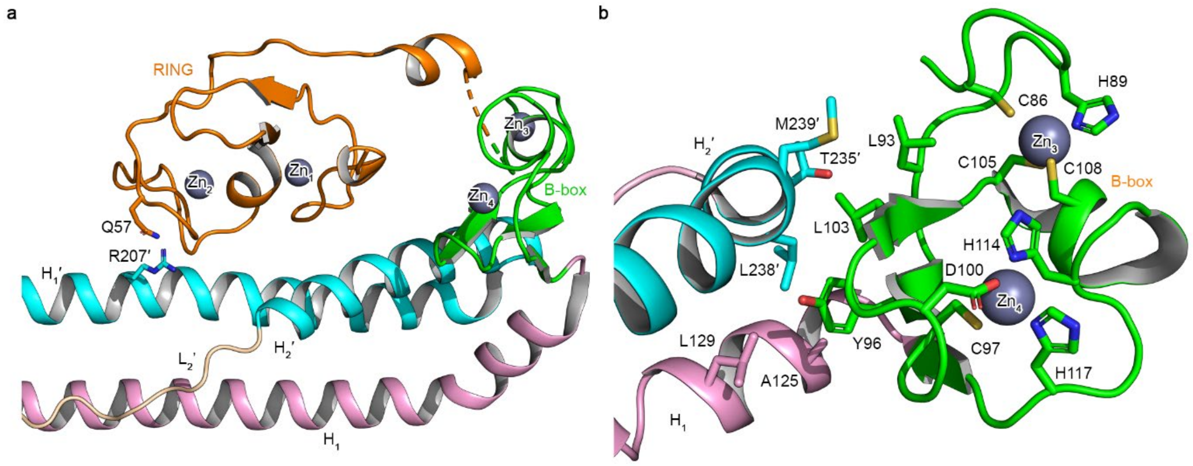

Supplementary Fig. 3| Cross-up view of the peripheral region of TRIM72.

a, Bent conformation of the RING domain. TRIM72 RING (orange), B-box (green), and CCD (H_1; pink, H_2; cyan) are shown in a ribbon diagram, and bound-zinc ions are shown as gray spheres. The suboptimal glutamine linchpin (Q57) and a nearby residue (R207') from the other CCD protomer are shown as stick models. The model is derived from the crystal structure of TRIM72 FL (4.6 Å). **b,** Hydrophobic interaction between the CCD and the B-box. The residues supplying hydrophobic interfaces or coordinating zinc ions (gray spheres) are represented in stick models. The model comes from the crystal structure of TRIM72 ΔRING (2.75 Å). All atoms are shown in the following colors: sulfur, yellow; nitrogen, blue; oxygen, red.

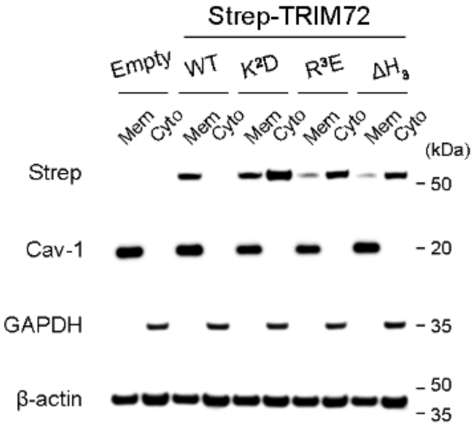

Supplementary Fig. 4| Membrane fractionation assay in C2C12 myoblast cells.

Subcellular fractionation of human TRIM72 in C2C12 myoblasts. The membrane (Mem) and cytosol (Cyto) fractions are indicated at the top. Strep-tagged TRIM72 was detected with an HRP-conjugated strep monoclonal antibody. A subcellular localization marker and loading control were detected with specific antibodies. Independent experiments were performed in triplicate. The quantified results are shown in **Fig. 2e**.

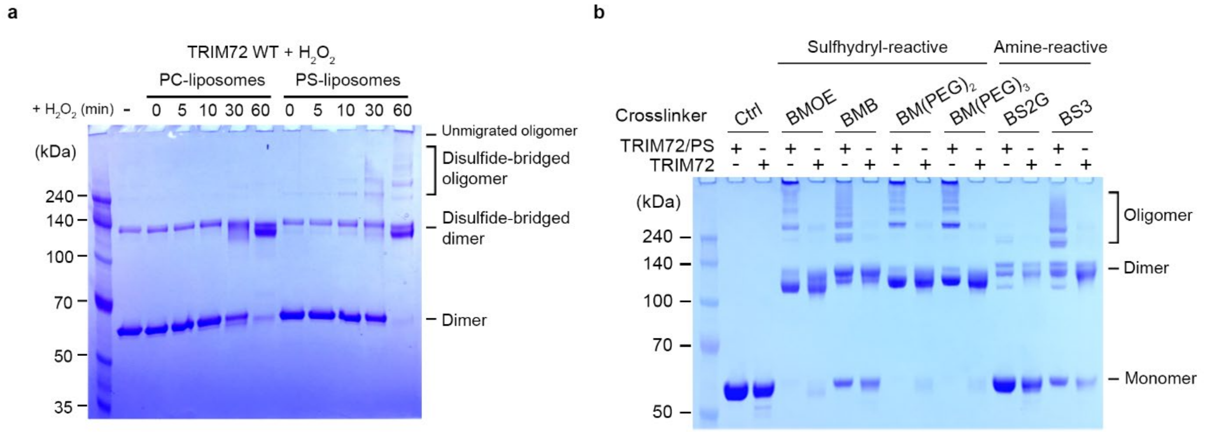

Supplementary Fig. 5| Crosslinking between TRIM72 in the presence of liposomes.

a, Time-dependent protein oxidation analysis of TRIM72. Hydrogen peroxide (H_2O_2) was used as an oxidizing reagent to detect the formation of disulfide bonds in the presence of PC- or PS-liposomes. **b,** Crosslinking between TRIM72 in the presence of PS-liposomes. The crosslinkers are indicated at the top. Ctrl: Control; TRIM72/PS: TRIM72-proteoliposomes. Note that the oligomerized bands are increased progressively only in lanes with PS-liposomes. Boiled samples were loaded onto 4-12% SDS–PAGE gels under nonreducing conditions.

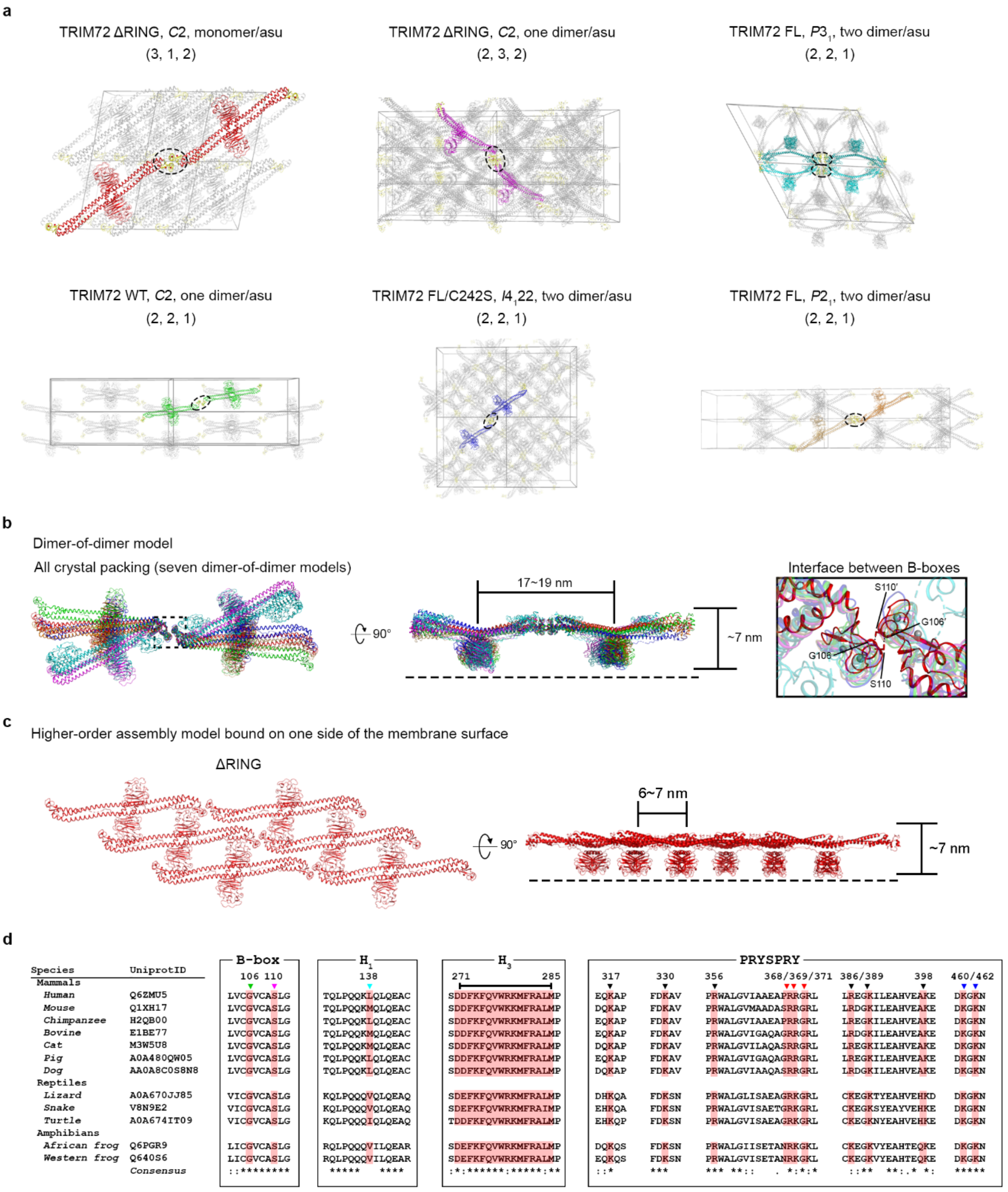

Supplementary Fig. 6| Structural analysis of the TRIM72 assembly in crystal packing.

a, Crystal packing of TRIM72. The constructs, space groups, and the number of molecules per asymmetric unit are shown at the top of each panel. The number of expanded unit cells is indicated in parentheses. The dimer-of-dimer model is highlighted with color in the crystal lattice. B-boxes are marked in yellow. Note that the B-boxes interacted with each other in all

crystal packings. The dashed ellipses indicate contacted B-boxes in dimer-of-dimer models. **b,** Superimposition of dimer-of-dimer models shows that PRYSPRY domains adopt the same orientation. The top view (left) and side view (middle) rotated by 90° are shown. The interfaces between B-boxes are also shown (right). The right panel is an enlarged dotted box. **c,** Symmetry expansion of the crystal packing of TRIM72 ΔRING (2.75 Å). The top view (left) and side view (right) rotated by 90° are shown. **d,** Sequence alignment of TRIM72 proteins from various species. Conserved residues for membrane binding and higher-order assembly in mammals, reptiles, and amphibians are shown as inverted triangles (top) and shaded in red. The number of residues corresponds to the sequence of mouse TRIM72. The TRIM72 sequences were aligned using Clustal Ω[5].

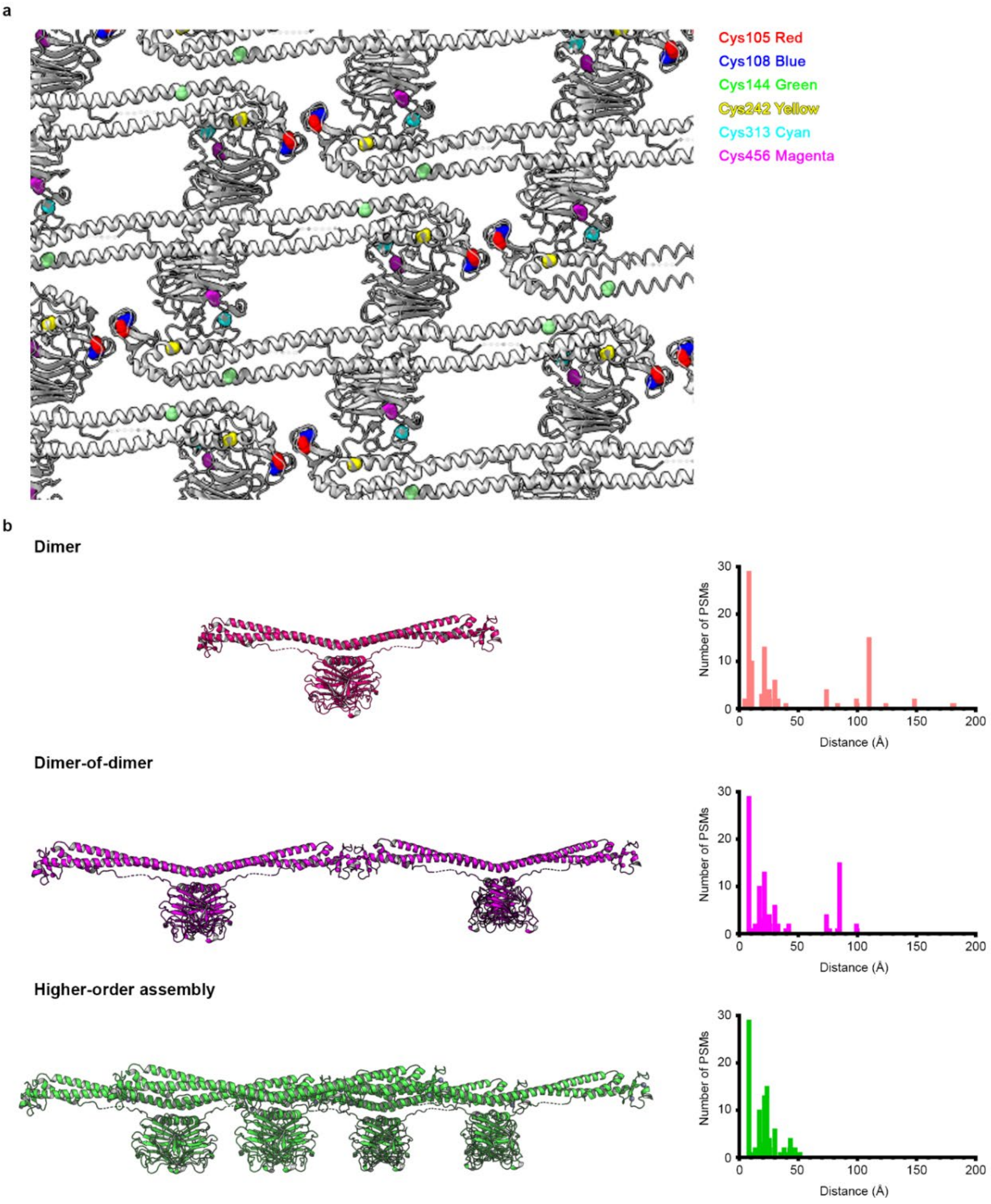

Supplementary Fig. 7| Crosslinking analysis of the higher-order TRIM72 assembly.

a, Coordinates of cysteine residues in the higher-order TRIM72 assembly. Each cysteine is represented with a colored ball (Cys105, red; Cys108, blue; Cys144, green; Cys242, yellow; Cys313, cyan; Cys456, magenta). **b,** Distances between crosslinked cysteines in each TRIM72 model. Compared to the dimer model or the dimer-of-dimer model, the higher-order assembly model shows an optimal distance range between crosslinked cysteines within 50 Å. The CLMS data are summarized in **Supplementary Data S2**. The experimental procedure is detailed in the **Methods**.

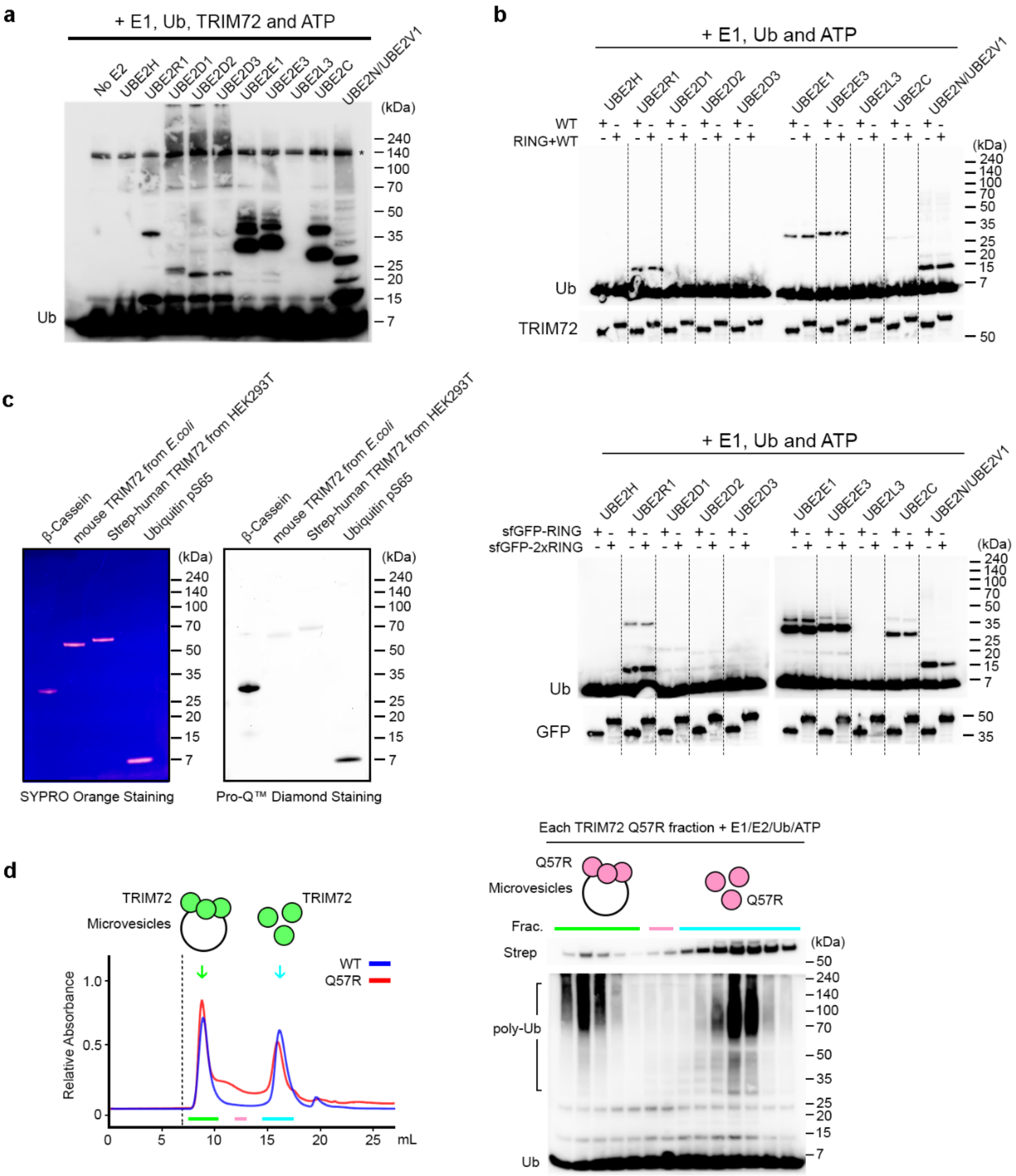

Supplementary Fig. 8| *In vitro* ubiquitination assay with TRIM72 RING constructs.

a, *In vitro* E2 screening assay. The TRIM72 protein with only UBE2Ds generated polyubiquitination chains. Ub-conjugated E2 is indicated by an asterisk (*). **b,** *In vitro* E2 screening with various TRIM72 constructs: WT and RING+WT (top), RING and 2×RING (bottom). **c,** SDS–PAGE analysis with phosphorylation-specific staining. Note that TRIM72 proteins purified from both bacterial and mammalian cells were not stained with phosphorylation-specific dyes. The total quantities of proteins were revealed by staining with

SYPRO Orange Protein Gel Stain (left). The phosphorylated proteins were revealed by staining with ProQ^{TM} Diamond Phosphoprotein Gel Stain (right). β-Catenin (Lane 1) and phosphorylated Ub at Ser65 (Lane 4) were the positive controls for the detection of phosphate groups. **d,** SEC elution profiles (left panel) of the microvesicle-bound (green arrow) and free-solution state (cyan arrow) of TRIM72 WT (blue line) and Q57R (red line) expressed in HEK293T cells. *In vitro* ubiquitination assay (right panel) with each fraction of TRIM72 Q57R expressed in HEK293T cells. The elution fractions are indicated with bars colored green, pink and cyan. The volume of each fraction was 0.5 mL. The void volume (≥ 5 MDa) is marked with a black dashed line. The details of the constructs are described in **Supplementary Table 1**.

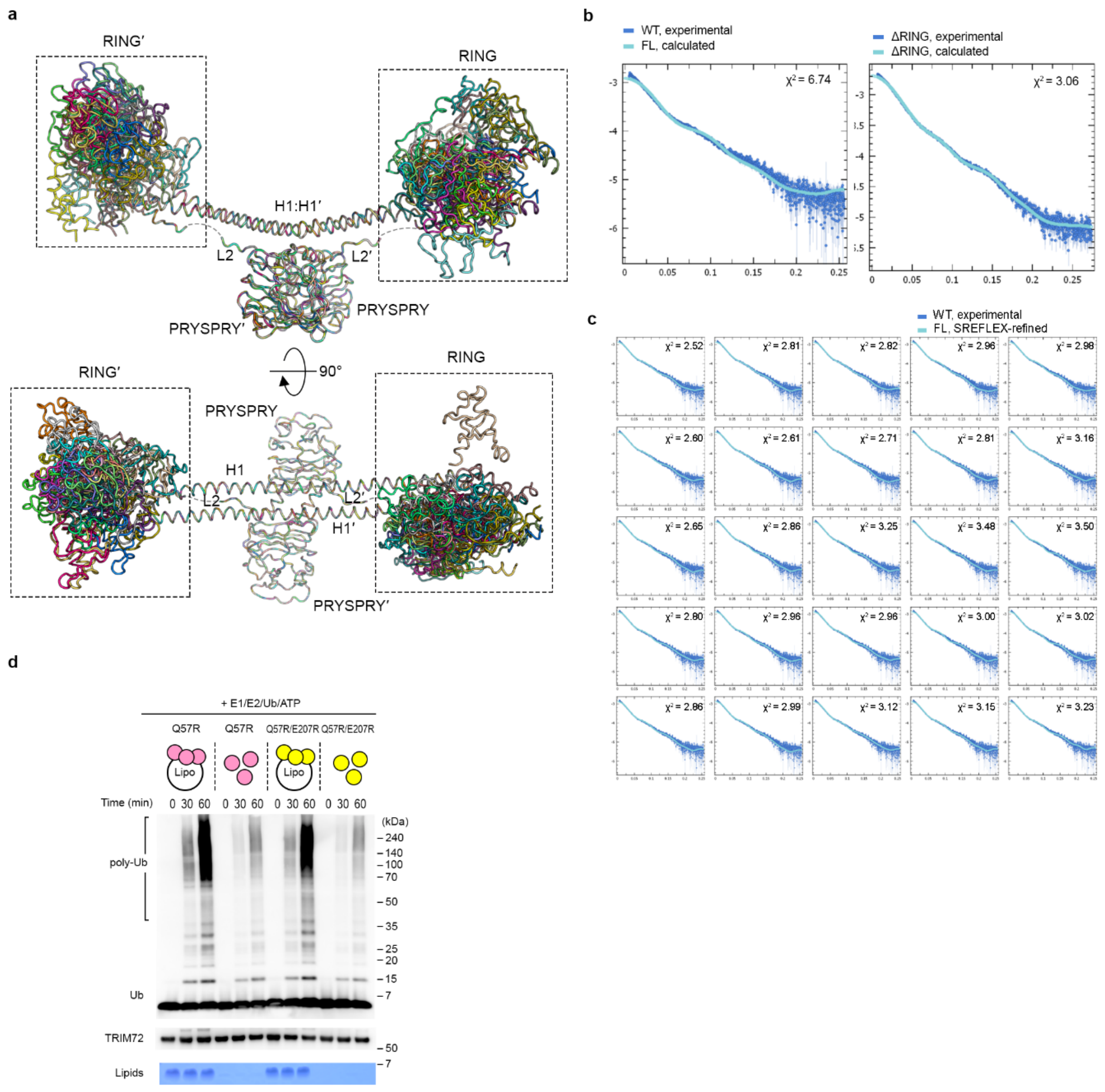

Supplementary Fig. 9| The RING domain is highly flexible.

a, Dynamic conformations of the RING domains. A total of 25 ensemble models were created based on the SREFLEX analysis. **b,** Comparison of the experimental (blue) and calculated (cyan) intensity plots for TRIM72 WT (left) and ΔRING (right). Theoretical SAXS profiles were calculated from the crystal structures of TRIM72 FL and ΔRING by CRYSOL software[6]. **c,** SREFLEX-derived SAXS profiles of TRIM72 WT corresponding to the ensemble Models (**a**). **d**, *In vitro* ubiquitination assay of TRIM72 Q57R and Q57R/E207R in the presence or absence of PS-liposomes. Polyubiquitination is shown as poly-Ub. Ub was detected with an anti-Ub antibody. TRIM72 was detected using an anti-TRIM72 antibody. Phospholipids were stained with Sudan black B. Schematic diagrams are shown in each panel for better communication of the results (Q57R, pink; Q57R/E207R, yellow).

Supplementary References

1. Lienlaf, M. et al. Contribution of E3-ubiquitin ligase activity to HIV-1 restriction by TRIM5alpha(rh): structure of the RING domain of TRIM5alpha. *J Virol* **85**, 8725-37 (2011).
2. Yudina, Z. et al. RING dimerization links higher-order assembly of TRIM5α to synthesis of K63-linked polyubiquitin. *Cell reports* **12**, 788-797 (2015).
3. Fletcher, A.J. et al. TRIM5alpha requires Ube2W to anchor Lys63-linked ubiquitin chains and restrict reverse transcription. *EMBO J* **34**, 2078-95 (2015).
4. Herkules, F. et al. Structural and functional asymmetry of RING trimerization controls priming and extension events in TRIM5alpha autoubiquitylation. *Nat Commun* **13**, 7104 (2022).
5. Sievers, F. et al. Fast, scalable generation of high-quality protein multiple sequence alignments using Clustal Omega. *Mol Syst Biol* **7**, 539 (2011).
6. Svergun, D., Barberato, C. & Koch, M.H.J. CRYSOL - A program to evaluate x-ray solution scattering of biological macromolecules from atomic coordinates. *Journal of Applied Crystallography* **28**, 768-773 (1995).
