# Supplementary material for: Structure and activation of the RING E3 ubiquitin ligase TRIM72 on the membrane
Source: Nat Struct Mol Biol. 2023 Sep 28;30(11):1695–706. doi: 10.1038/s41594-023-01111-7 (PMC10643145; doi:10.1038/s41594-023-01111-7)
Supplement: Supplementary file 2 — Reporting Summary [file 41594_2023_1111_MOESM2_ESM.pdf]

nature research

Corresponding author(s): Hyun Kyu Song

Last updated by author(s): Jul 5, 2023

Reporting Summary

Nature Research wishes to improve the reproducibility of the work that we publish. This form provides structure for consistency and transparency in reporting. For further information on Nature Research policies, see our Editorial Policies and the Editorial Policy Checklist.

Statistics

For all statistical analyses, confirm that the following items are present in the figure legend, table legend, main text, or Methods section.

n/a	Confirmed	
☐	☒	The exact sample size (*n*) for each experimental group/condition, given as a discrete number and unit of measurement
☐	☒	A statement on whether measurements were taken from distinct samples or whether the same sample was measured repeatedly
☐	☒	The statistical test(s) used AND whether they are one- or two-sided<br>*Only common tests should be described solely by name; describe more complex techniques in the Methods section.*
☒	☐	A description of all covariates tested
☒	☐	A description of any assumptions or corrections, such as tests of normality and adjustment for multiple comparisons
☐	☒	A full description of the statistical parameters including central tendency (e.g. means) or other basic estimates (e.g. regression coefficient) AND variation (e.g. standard deviation) or associated estimates of uncertainty (e.g. confidence intervals)
☐	☒	For null hypothesis testing, the test statistic (e.g. *F*, *t*, *r*) with confidence intervals, effect sizes, degrees of freedom and *P* value noted<br>*Give P values as exact values whenever suitable.*
☒	☐	For Bayesian analysis, information on the choice of priors and Markov chain Monte Carlo settings
☒	☐	For hierarchical and complex designs, identification of the appropriate level for tests and full reporting of outcomes
☒	☐	Estimates of effect sizes (e.g. Cohen's *d*, Pearson's *r*), indicating how they were calculated

Our web collection on statistics for biologists contains articles on many of the points above.

Software and code

Policy information about availability of computer code

Data collection	Crystallographic and SAXS data collection: software developed in each synchrotron beamline in PAL-5C, PF-NE3, PF-NW12, SP-44XU and PAL-4C, PF-10C, respectively; MALS data collection; ASTRA V; Liposome binding analysis using flow cytometry: BD Accuri C6; SPR: Biacore T100 Chromatography; UNICORN 7.0; Western blot: ImageQuant LAS 4000 mini; Cryo-EM: FEI EPU; Cryo-ET: Tomography 4.0; Mass spectrometry: Q exactive mass spectrometer.
Data analysis	Crystallographic data analysis: HKL-2000 v722 and CCP4 suite v8.0; MR and data refine: PHENIX package; Model building: Coot-0.9.8; SAXS data analysis: ATSAS package 3.2.1, ATSAS online and GraphPad Prism 6; Stucture visualization: PyMol 2.5.4 and UCSF ChimeraX 1.5; MALS analysis: ASTRA V and GraphPad Prism 6; Liposome analysis: Dynamics Pro, BD Accuri C6 Plus and GraphPad Prism 6; SPR data analysis: BIAevaluation and GraphPad Prism 6; Chromatography; UNICORN 7.0 and GraphPad Prism 6; Western blot analysis: ImageJ and GraphPad Prism 6; Cryo-ET reconstruction: IMOD 4.11, Dynamo package 1.1.532 and RELION4.0; Multiple Sequence alignment: Bioedit, PROMALS3D and Clustal Omega; Cross-linking analysis: pLink 2 and Xwalk; Mass spectrometry: Proteome Discoverer platform; Gene ontology analysis: DAVID software.

For manuscripts utilizing custom algorithms or software that are central to the research but not yet described in published literature, software must be made available to editors and reviewers. We strongly encourage code deposition in a community repository (e.g. GitHub). See the Nature Research guidelines for submitting code & software for further information.

Data

Policy information about availability of data

All manuscripts must include a data availability statement. This statement should provide the following information, where applicable:

- Accession codes, unique identifiers, or web links for publicly available datasets
- A list of figures that have associated raw data
- A description of any restrictions on data availability

The protein data bank (PDB) with the accession codes 7XV2 7XYY, 7XYZ, 7XZ0 7XZ1 and 7XZ2 for crystal structures of TRIM72. The details were described in the extended data table 2. The PDB code 3KB5 was used for structure determination. The PDB codes 2LM3, 7W0T, 2IWG, 7JL0 and 7JL1 were used for structural analysis. The small angle scattering biological data bank (SASDB) with accession codes SASDK86 and SASDK96 for TRIM72 wild-type and ΔRING, respectively. The Electron Microscopy Database (EMDB) with the accession codes EMD-31139, EMD-31151, EMD-33569 and EMD-33582 for structures of the reconstituted proteoliposome of TRIM72 WT or M138R. The Proteomics Identifications Database (PRIDE) with the accession codes PXD024946 and PXD024978 for cross-linking assay and gene ontology analysis, respectively. All available data will be released upon publication.

Field-specific reporting

Please select the one below that is the best fit for your research. If you are not sure, read the appropriate sections before making your selection.

☒ Life sciences ☐ Behavioural & social sciences ☐ Ecological, evolutionary & environmental sciences

For a reference copy of the document with all sections, see nature.com/documents/nr-reporting-summary-flat.pdf

Life sciences study design

All studies must disclose on these points even when the disclosure is negative.

Sample size	For solving crystal structures, over 1,000 crystal conditions were tested and screened. For cryo-ET, over 100 grids were used for optimization.
Data exclusions	No data were excluded from the analysis.
Replication	All experiments were performed at least twice (mostly three times) and were replicated successfully.
Randomization	For the 3D reconstruction of subtomogram averaging and R-free calculation of the crystal structure, samples were randomly divided into halves. Other experiments are not involved in randomization.
Blinding	Blinding is not applicable for this study, which were structural and biochemical studies.

Reporting for specific materials, systems and methods

We require information from authors about some types of materials, experimental systems and methods used in many studies. Here, indicate whether each material, system or method listed is relevant to your study. If you are not sure if a list item applies to your research, read the appropriate section before selecting a response.

Materials & experimental systems

n/a	Involved in the study	
☐	☒	Antibodies
☐	☒	Eukaryotic cell lines
☒	☐	Palaeontology and archaeology
☒	☐	Animals and other organisms
☒	☐	Human research participants
☒	☐	Clinical data
☒	☐	Dual use research of concern

Methods

n/a	Involved in the study	
☒	☐	ChIP-seq
☒	☐	Flow cytometry
☒	☐	MRI-based neuroimaging

Antibodies

Antibodies used	The antibody name (with clone name when available), supplier name, catalog number, lot number and dilution factor are as follows;<br>anti-MG53 antibody, Abcam, cat. No. ab154238, Lot No. GR196951-2, 1:5,000.<br>anti-Ubiquitin antibody (P4D1), Santa Cruz, cat. No. sc-8017, Lot No. J0716, 1:1,000.<br>anti-Ubiquitin antibody (VU-1), LifeSensors, cat. No. VU101, Lot No. AB40627.005, 1:1,000.<br>StrepMAB-Classic HRP, IBA, cat. No. 2-1509-001, Lot No. 1509-0075, 1:20,000.<br>anti-Caveolin-1 antibody, CST, cat. No. 3238S, Lot No. 3, 1:1,000.<br>anti-GAPDH antibody (0411), Santa Cruz, cat. No. sc-47724, Lot No. K0615, 1:1,000.<br>anti-GFP antibody (B-2), Santa Cruz, cat. No. sc-9996, Lot No. H1122, 1:5,000.

anti ATP1A1 (C464.6), Santa Cruz, cat. sc-21712, Lot No. I1820, No, 1:1,000.
anti-beta actin antibody (C4), Santa Cruz, cat. No. sc-47778, Lot. No. L1616, 1:5,000.
anti-rabbit IgG, HRP-linked antibody, CST, cat. No. 7074S, Lot. No. 27, 1:10,000.
mouse IgG kappa BP-HRP, Santa Cruz, cat. No. sc-516102, Lot. No. I1317, 1:10,000.

Validation

The commercial antibodies employed in our study were validated by the manufacturers. The manufacturer's on-line data sheet for each antibody is located at the following web address;
anti-MG53 antibody; rabbit poylclonal IgG; suitable for western blot; reacting with human; https://www.abcam.com/mg53-antibody-ab154238.html
anti-Ubiquitin antibody (P4D1); mouse monoclonal IgG; suitable for broad application including western blot; reacting with human, mouse, rabbit and fruit fly; Citation: Cell (1996) 84:852-62.; https://www.scbt.com/p/ubiquitin-antibody-p4d1
anti-Ubiquitin antibody (VU-1); mouse monoclonal IgG; suitable for western blot and immunohistochemistry; reacting with human and mouse; Citation: Nature (2002) 416:648-53.; https://lifesensors.com/product/vu101-anti-ubiquitin-antibody-mab-clone-vu-1
StrepMAP-Classic HRP; mouse monoclonal IgG; suitable for western blot, reacting with Strep-tag II and Twin-Strep-tag; Citation: Nat. Commun. (2020) 11:2251.; https://www.iba-lifesciences.com/strepmab-classic-hrp-conjugate/2-1509-001
anti-Caveolin-1 antibody; rabbit polyclonal IgG; suitable for western blot; reacting with human, mouse and hamster; Citation: J Biol Chem (1998) 273:5419-22.; https://www.cellsignal.com/products/primary-antibodies/caveolin-1-antibody/3238
anti-GAPDH antibody (0411); mouse monoclonal IgG; suitable for western blot, reacting human; Citation: Nat. Neurosci. (2019) 22:1235-1247.; https://www.scbt.com/p/gapdh-antibody-0411
anti-beta actin antibody (C4); mouse monoclonal IgG; suitable for general application including western blot; reacting with human, mouse, rat and bovine; Citation: Cell (1995) 81:53-62.; https://www.scbt.com/p/beta-actin-antibody-c4
anti-GFP antibody (B-2); mouse monoclonal IgG; suitable for broad application including western blot; reacting with amino acids 1-238 representing full-length GFP and its variants of Aequorea victoria origin; Citation: Science (1994) 263:802-5.; https://www.scbt.com/p/gfp-antibody-b-2
anti ATP1A1 (C464.6); mouse monoclonal IgG; suitable for western blot; reacting with broad range of species including human; Citation: J Biol Chem (2001) 276:20370-8.; https://www.scbt.com/p/na-k-atpase-alpha1-antibody-c464-6
anti-rabbit IgG, HRP-linked antibody; goat anti-rabbit IgG; suitable for western blot; reacting with rabbit polyclonal and monoclonal antibodies; https://www.cellsignal.com/products/secondary-antibodies/anti-rabbit-igg-hrp-linked-antibody/7074
mouse IgG kappa BP-HRP; mouse IgG kappa binding protein; suitable for western blot; reacting with mouse IgG kappa chain; https://www.scbt.com/p/m-igg-kappa-bp-hrp

Eukaryotic cell lines

Policy information about cell lines

Cell line source(s)	HEK293T cells (CRL-3216, ATCC) and C2C12 cells (CRL-1772, ATCC).
Authentication	Commercial cell lines were authenticated by manufacturers. C2C12 cells were further validated by muscle differentiation.
Mycoplasma contamination	Tested negative for Mycoplasma contamination.
Commonly misidentified lines (See ICLAC register)	No commonly misidentified cell lines have been used in this study.
