# Supplementary material for: Structure and activation of the RING E3 ubiquitin ligase TRIM72 on the membrane
Source: Nat Struct Mol Biol. 2023 Sep 28;30(11):1695–706. doi: 10.1038/s41594-023-01111-7 (PMC10643145; doi:10.1038/s41594-023-01111-7)
Supplement: Supplementary file 10 — Unprocessed western blots for Supplementary Figs. 4, 8a–d and 9d. [file 41594_2023_1111_MOESM10_ESM.pdf]

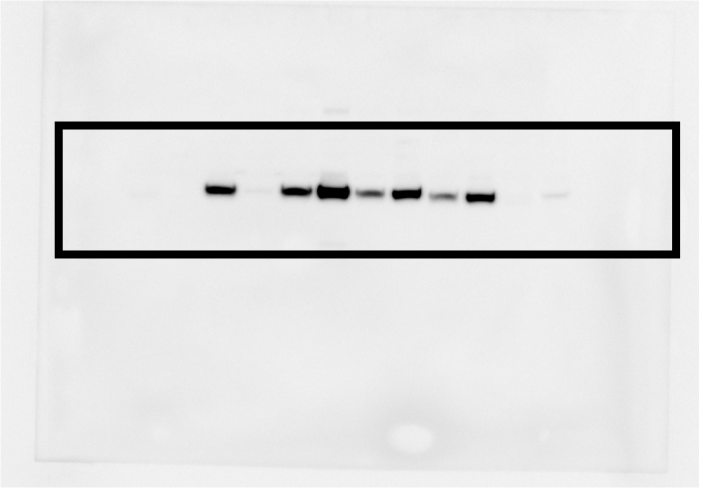

Anti-Strep

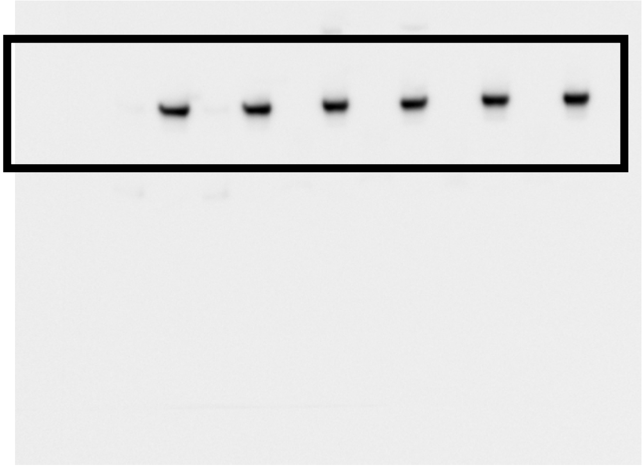

Anti-GAPDH

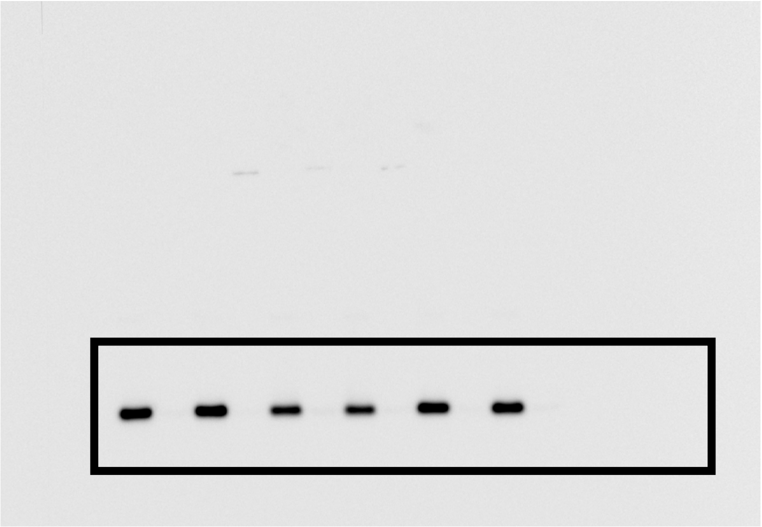

Anti-Cav-1

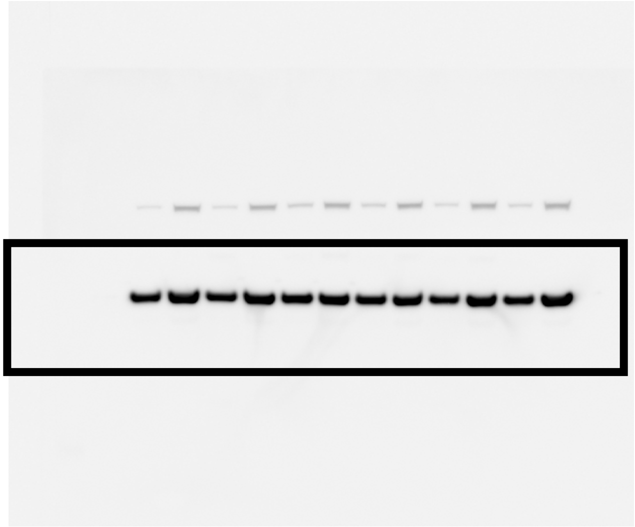

Anti-β-actin

Supplementary Fig. 4

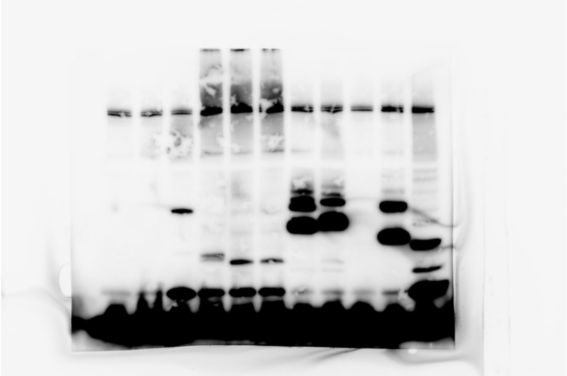

Anti-Ub

Supplementary Fig. 8a

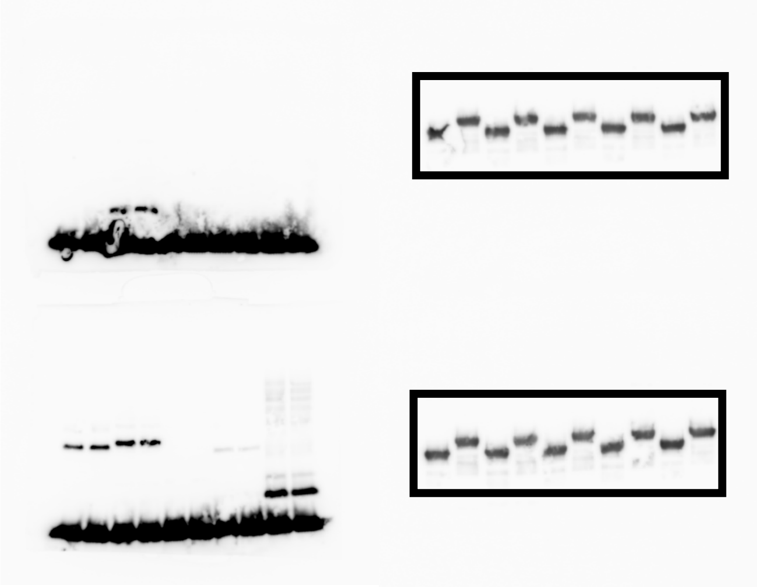

Anti-Ub

Anti-TRIM72

Supplementary Fig. 8b

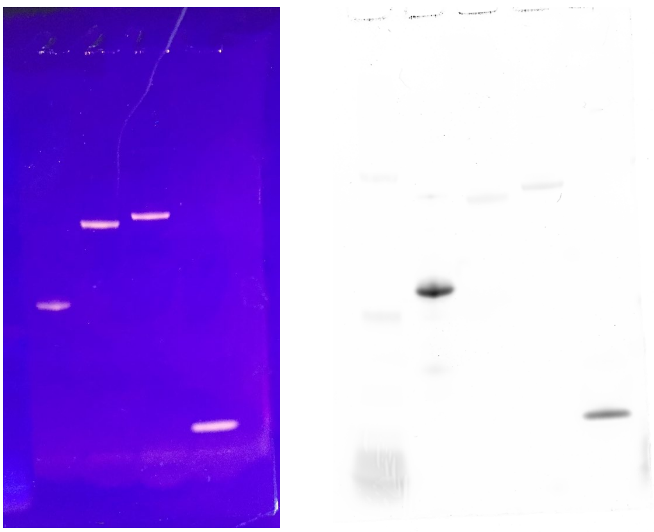

SYPRO Orange

ProQ Diamond

Supplementary Fig. 8c

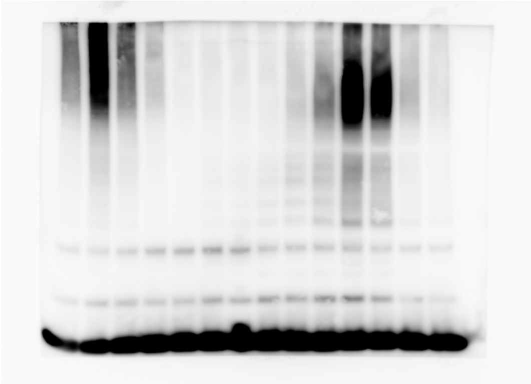

Anti-Ub

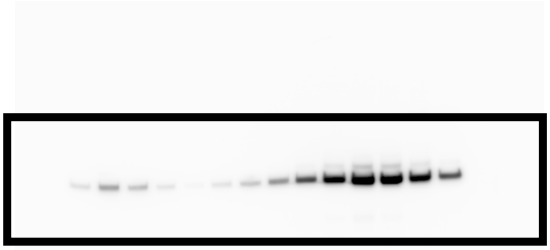

Anti-Strep

Supplementary Fig. 8d

Anti-Ub

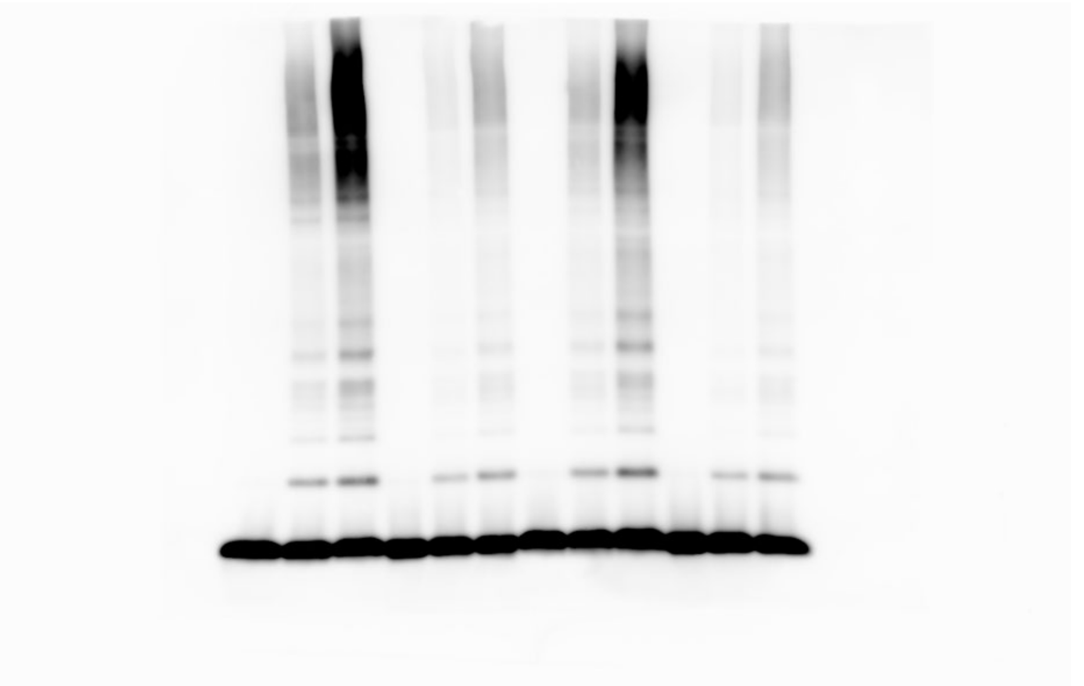

Anti-TRIM72

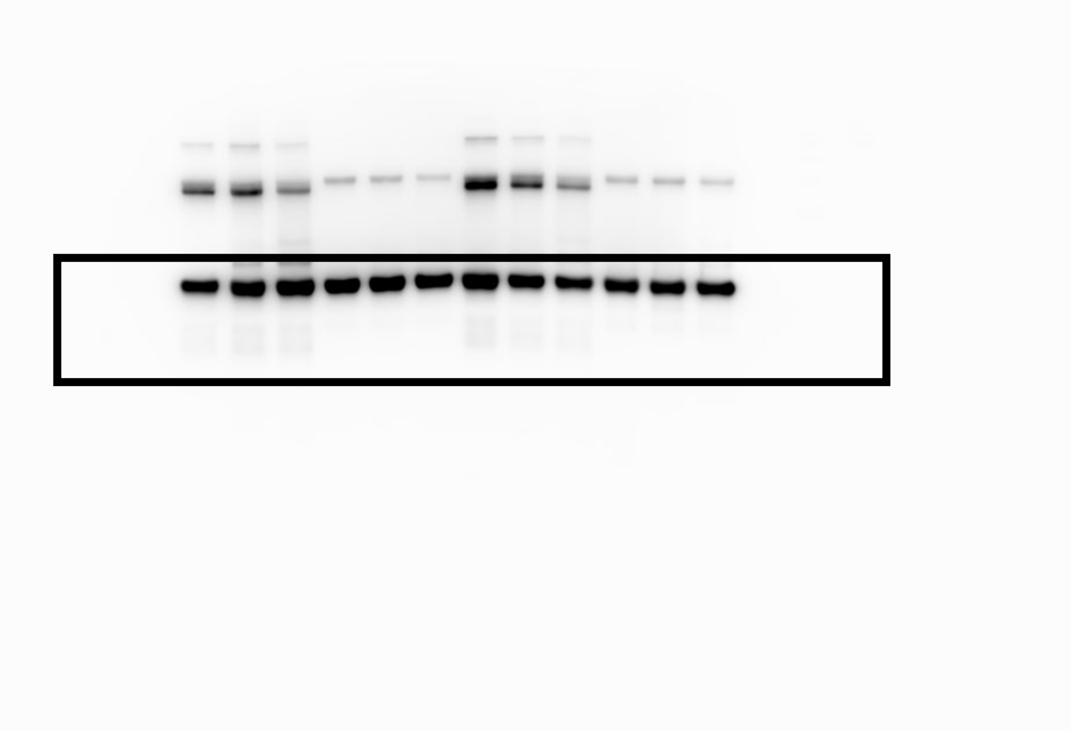

Sudan Black B

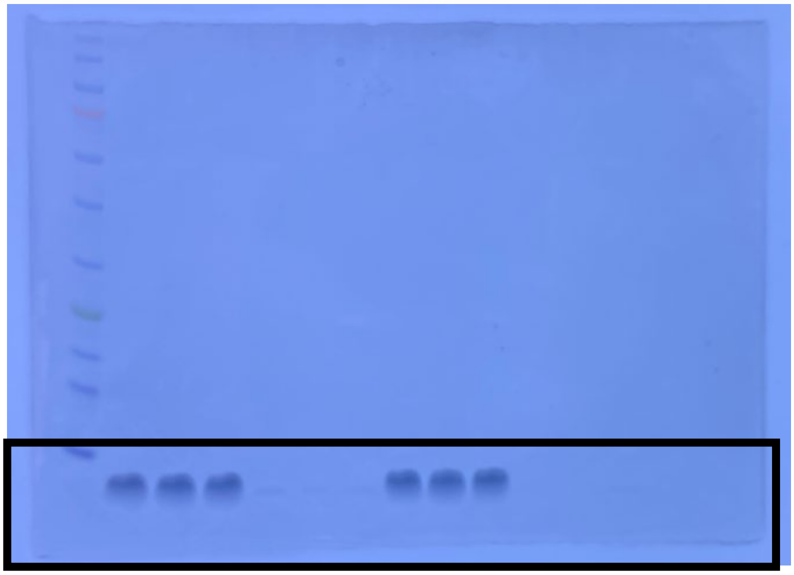

Supplementary Fig. 9d
