# Supplementary figures and images for: Structure and activation of the RING E3 ubiquitin ligase TRIM72 on the membrane
Source: Nat Struct Mol Biol. 2023 Sep 28;30(11):1695–706. doi: 10.1038/s41594-023-01111-7 (PMC10643145; doi:10.1038/s41594-023-01111-7)

Anti-TRIM72

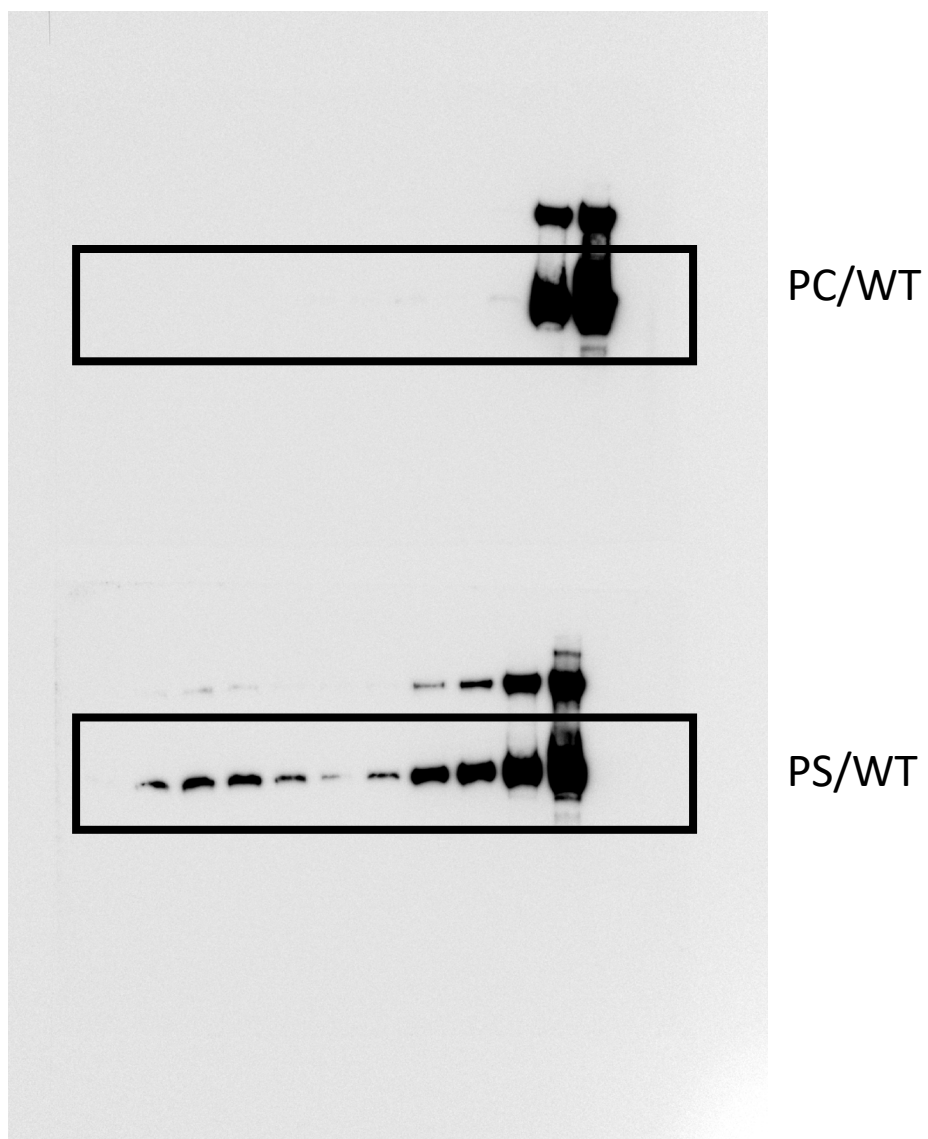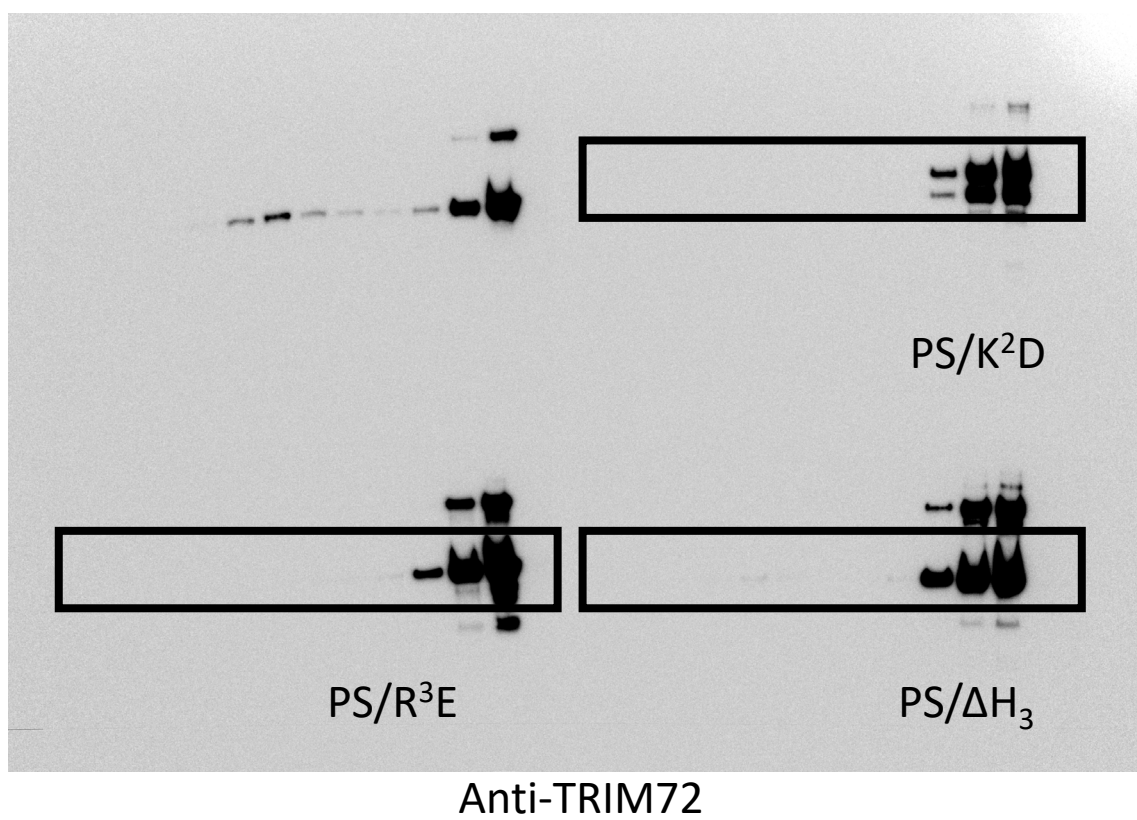

Fig. 2d

Supplement: Supplementary file 12 — Unprocessed western blots. [file 41594_2023_1111_MOESM12_ESM.pdf]

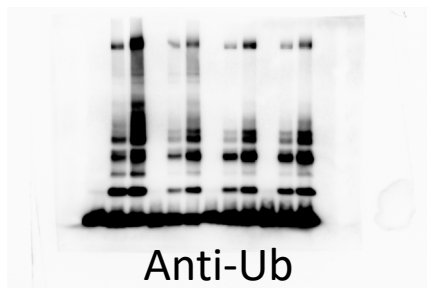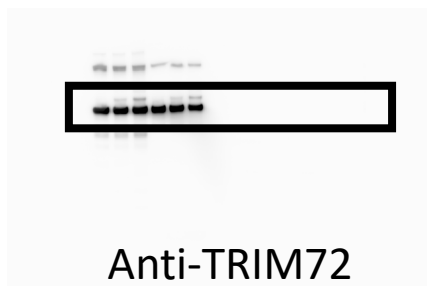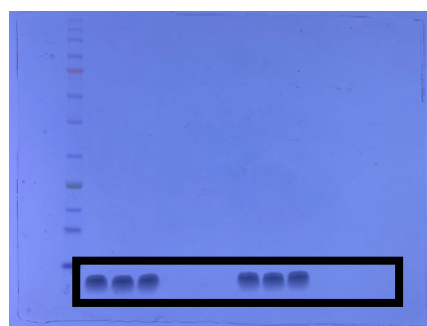

Fig. 4a

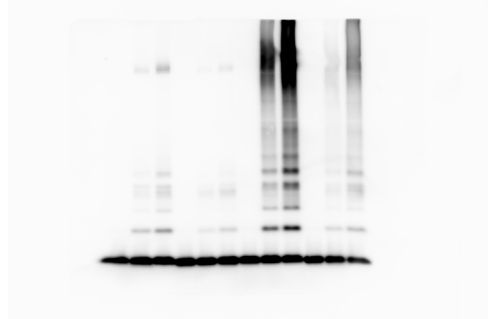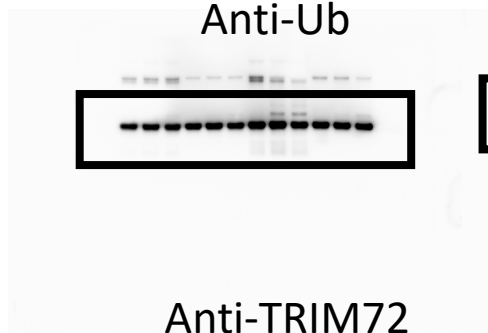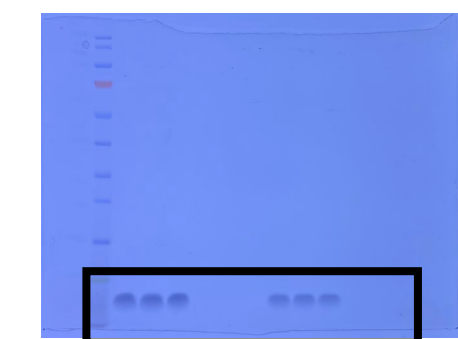

Fig. 4b

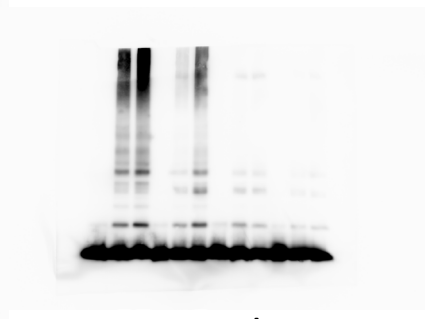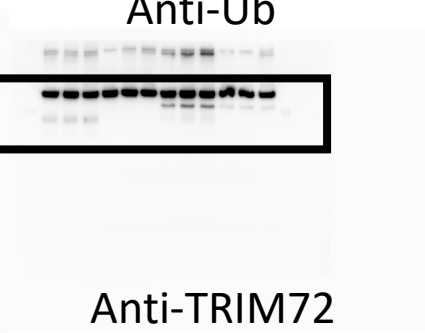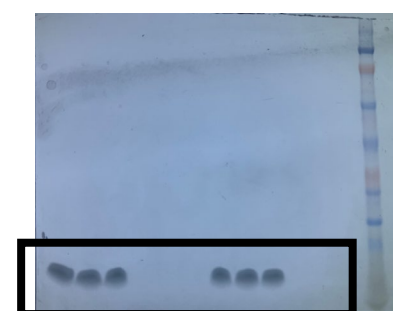

Fig. 4c

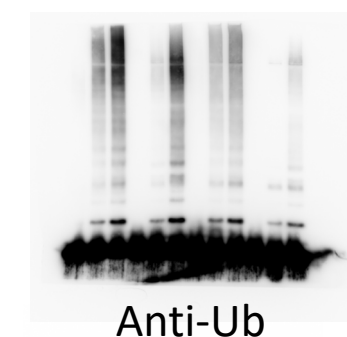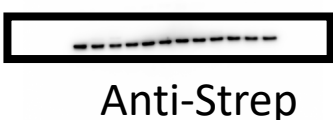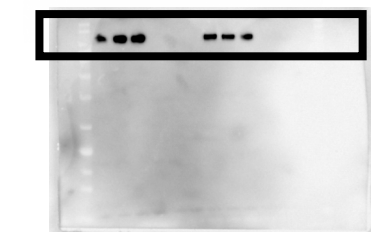

Fig. 4d

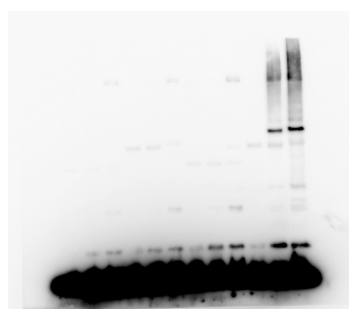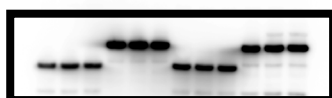

Fig. 4e

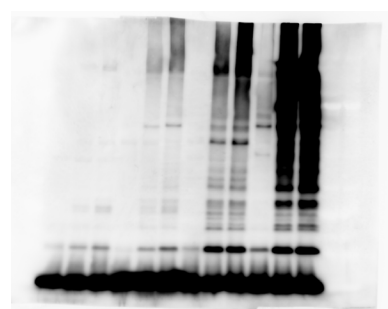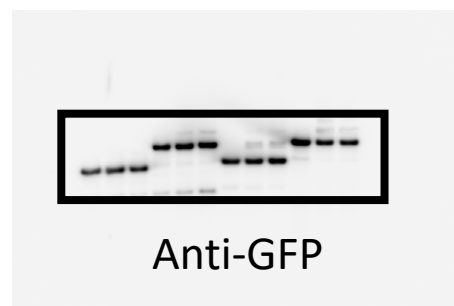

Fig. 4f

Supplement: Supplementary file 14 — Unprocessed western blots. [file 41594_2023_1111_MOESM14_ESM.pdf]

- Ca      + Ca      - Ca      + Ca  
- EGTA   - EGTA   + EGTA   + EGTA

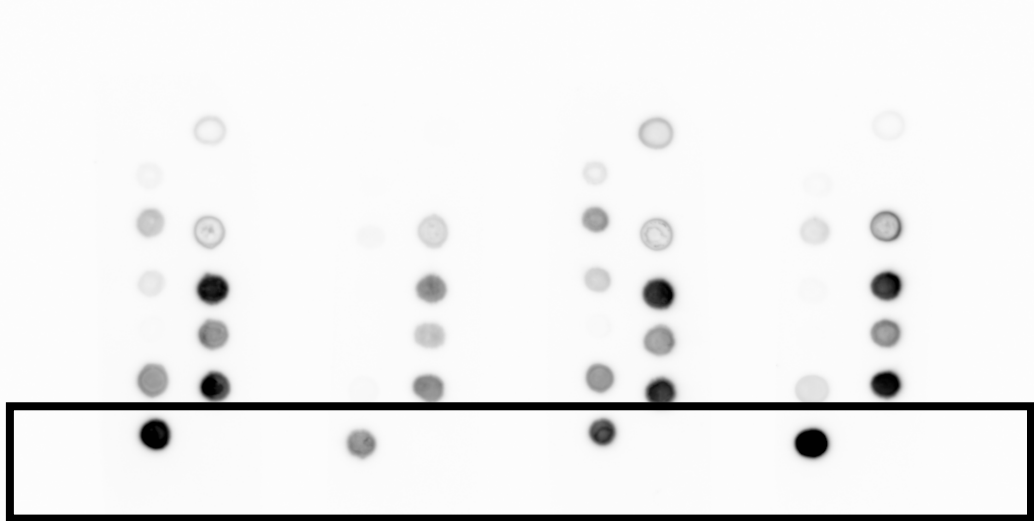

Anti-TRIM72

Supplement: Supplementary file 15 — Unprocessed western blots. [file 41594_2023_1111_MOESM15_ESM.pdf]

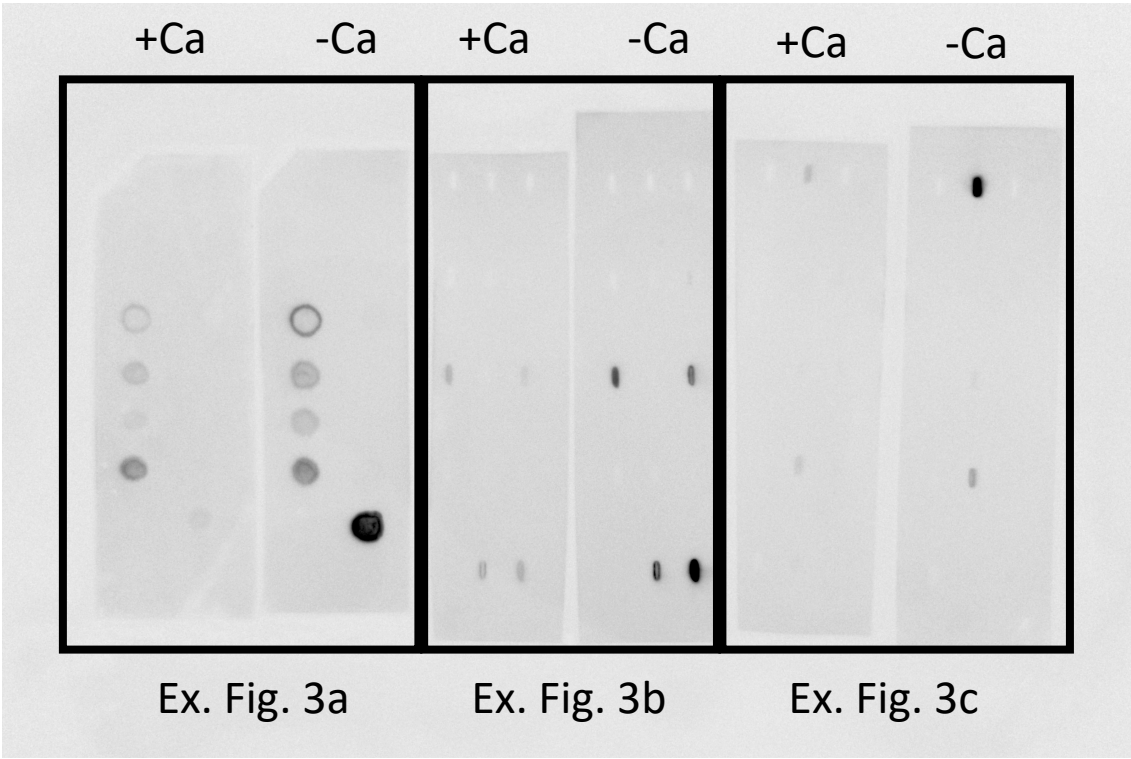

Anti-TRIM72

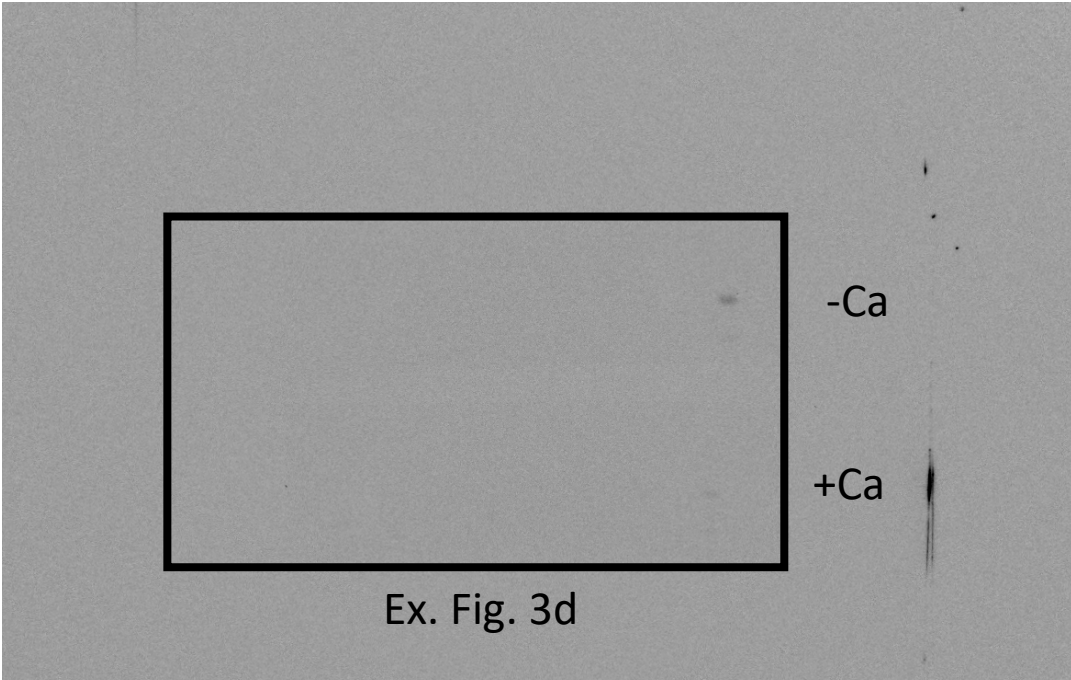

Anti-TRIM72

Supplement: Supplementary file 19 — Unprocessed western blots. [file 41594_2023_1111_MOESM19_ESM.pdf]
